# Impact of Intravitreal Injection of Bevacizumab (Avastin) on Rabbit's Choroid and Retina

**DOI:** 10.4103/0974-9233.51995

**Published:** 2008

**Authors:** Sherif Karawya, Dalia G. Said, Mostafa M. Salaheldin, Iman Zaky

**Affiliations:** 1 From the Ophthalmology Department, Research Institute of Ophthalmology, Cairo, Egypt; 2 From the Pathology Department, Research Institute of Ophthalmology, Cairo, Egypt

**Keywords:** Bevacizumab, intravitreal injection, chorio-capillaris, retinal toxicity, vascular endothelial growth factor

## Abstract

**Aim:**

The aim of the study was to evaluate the impact of intravitreal injection of bevacizumab (Avastin) on chorio-capillaris permeability as well as structure changes in the choroid and the retina of pigmented rabbits.

**Material and Methods:**

The study included 10 pigmented rabbits (20 clinically free eyes) ranged in weight between 1.2 and 2 kg (mean 1.7±0.05). The rabbits were subjected to intravitreal injection of 5 mg, 0.1mg Avastin in the right eyes (10eyes), while the left eyes (10eyes) were injected with equal volumes of balanced salt solution. 1 week later, Clinical examination and fundus fluorescein angiography (FFA) were done. Histological examination was performed on specimens of retina & choroid of Avastin & BSS injected eyes of sacrificed rabbits using light microscopy (LM) & transmission electron microscopy (TEM). Results were recorded and compared

**Results:**

Post injection clinical examination of the eyes showed no abnormality of cornea, lens, vitreous and fundus. **FFA** showed remarkable decrease in background chorio-capillaris fluorescence in 7 eyes (70 percent) injected with Avastin as compared with eyes injected with BSS. No change was observed in regards to retinal vasculature, or abnormal dye leak. **LM** examination: specimens from Avastin group were evaluated in comparison to control eyes Treated eyes exhibited the same microscopic appearance in most specimens (8/10, 80 percent). The chorio-capillaris layer showed elongated, stretched monolayer of capillaries with flat, elongated endothelial cell lining. The laminae showed closely packed RBCs arranged in a monolayer with ribbon like shape. The surrounding interstitial tissue showed stretched, elongated & compact collagen fibers. The RPE cells were tightly adherent to each other with prominent nuclei. The different retinal layers were in concomitance with the control specimens, however mild to moderate disruption of photoreceptor outer segments together with mild vacuolization in the ganglion cell layer were seen. TEM examination of both control and treated specimens confirmed the findings recorded by LM. The endothelial cell limning of the choriocapillaris exhibited reduced fenestrations in between the cells. TEM also highlighted the compact lamellae of collagen fibers. The RPE cells showed remarkable increase in the number of mitochondria and prominent endoplasmic reticulum. Variable sized melanosomes were also seen

**Conclusion:**

Though single intravitreal injection of Avastin does not cause appreciable histological changes in rabbit retina and choroid, yet, it imposes definite effect on choriocapillaris permeability as evidenced by FFA and ultra structural changes. Repeated intravitreal injections might alter the hemostasis of the chorio-capillaris RPE complex.

The choroid vessels between the retina and the sclera of the eye originate from mesodermal tissue surrounding the newly formed optic cup early in development. The choroid endothelial cells are derived from the periocular mesenchyme. It is thought that the choroid vessels are induced by the RPE during development. Vascular endothelial growth factor (VEGF) might be a critical mediator of these RPE functions.^1^ In tissue culture, multiple retinal cell types make VEGF and increase its production when the microenvironment is hypoxic.^2^–^3^ Angiogenesis is a highly complex and coordinated process requiring the sequential activation of a series of receptors in endothelial and mural cells. The data supporting a causal role for VEGF in ocular neovascularization are extensive.^4^–^7^

Among the leading causes of blindness are retina and choroid diseases manifesting abnormal vessel permeability and growth. The advent of anti VEGF treatments marks a major advancement in the treatment of these eye diseases.^8^,^9^

Little is known of the role of VEGF in the maintenance of adult ocular vasculature. VEGF is produced by human differentiated RPE cells and may be involved in paracrine signaling between RPE and chorio-capillaris.^10^ Indirect evidence suggests that VEGF may be trophic for the chorio-capillaris and required for the maintenance of the chorio-capillaris fenestrae.^8^

The aim of the present study was to evaluate the impact of intravitreal injection of bevacizumab (Avastin) on chorio-capillaris permeability as well as structure changes in the choroid and the retina of pigmented rabbits.

## Material and Methods

The study included 10 pigmented rabbits (20 clinically free eyes) ranged in weight between 1.2 and 2 kg (mean 1.7±0.05). The rabbits were subjected to intravitreal injection of 5 mg, 0.1mg Avastin in the right eyes (10 eyes), while the left eyes (10 eyes) were injected with equal volumes of balanced salt solution (BSS).

The animals were examined daily and animals showing anterior segment pathology or infection were excluded from the study. FFA was done 1 week after the Intravitreal injection followed by sacrificing the animals with subsequent histopathological study of choroid & retina.

### Intravitreal Injection

The animals were hypnotized by intra-muscular injection of 1ml (50mg) ketamin. Benoxinate hydrochloride 1% local anesthetic eye drops were installed into the conjunctival sac.

Paracentesis was done to prevent reflux of the injectant. Thereafter, 0.1 ml of the solution (Avastin or BSS) was injected into the vitreous cavity, using 30 caliber needles on insulin syringe. Injection site was selected 1mm outside the limbus.

### Fundus Fluorescein Angiography (FFA)

The pupil of each eye was dilated with tropicamide hydrochloride 1% eye drops. The animals were sedated by a single I.M injection of promazine hydrochloride (2.33 mg/kg). The procedure started by injecting 1 ml flourescein solution (10 %) into the ear penna venule. Sequential photography of the fundus was done as soon as the dye reached the retinal circulation, after activation of the exciter and barrier filters using a Topcon TRC 50 camera. The angiographs were studied primarily to assess degree of background choroid fluorescence as an indicator of chorio-capillaris permeability. Fluorescein angiographs were randomly arranged and assessed by the three contributing retina specialists individually.

### Histopathology

The animals were sacrificed (using an over dose of IV phenobarbitone). The eyes were enucleated & immediately bisected and fixed overnight with 4% gluteraldhyde in phosphate buffer PH 7.3. Sections of the posterior segment (2×2) were taken 2mm inferior to the optic disc, and post fixed with 1.3% osmium tetroxide in phosphate buffer PH 7.3, for 3 hours, embedded in Epon after dehydration in a graded series of acetones. Later, semi-thin Sections were obtained, stained with Toluidine blue stain and examined by light microscopy (LM). Further ultrathin sections were cut and double stained with Urynit acetate and Lead citrate and examined with Transmission Electron Microscope (TEM).

LM and TEM examination aimed to evaluate primarily the chorio-capillaris layer, in addition to RPE cells, and the neurosensory retina.

## Results

Post injection clinical examination of the eyes showed no abnormality of cornea, lens, vitreous and fundus.

**Figure 1:**
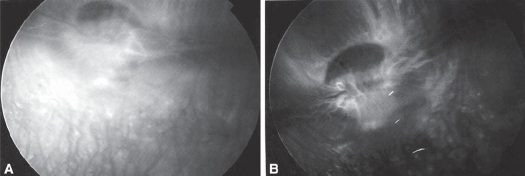
(A) Fundus fluorescein angiography (FFA) of control eye showing obvious background choroid fluorescence. (B) FFA of treated eye showing generalized decrease in choroid fluorescence.

Concerning FFA, there was an agreement of remarkable decrease in background chorio-capillaris fluorescence in 7 eyes (70 percent) injected with Avastin as compared with eyes injected with BSS. No change was observed as regards retinal vasculature, or abnormal dye leak (Figs 1a & b).

**Figure 2:**
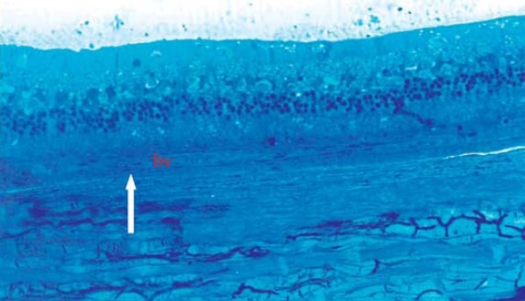
Light micrograph of control specimen showing choroid blood vessel (bv) with average diameter and prominent endothelial cell lining (arrow). The vessels are engorged with RBCs. The overlying retina appears with its different layers, (Toluidine blue × 250).

**Figure 3:**
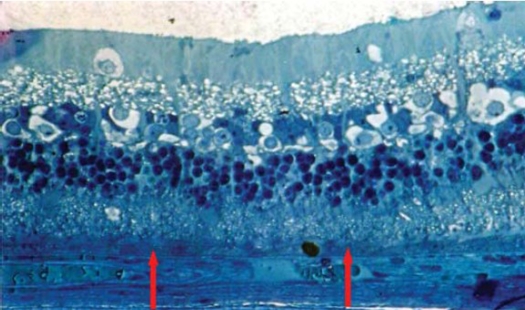
Light micrograph of control specimen with higher magnification. The RPE cells are plump-shaped and regularly arranged (arrow), (Toluidine blue × 500).

**Figure 4:**
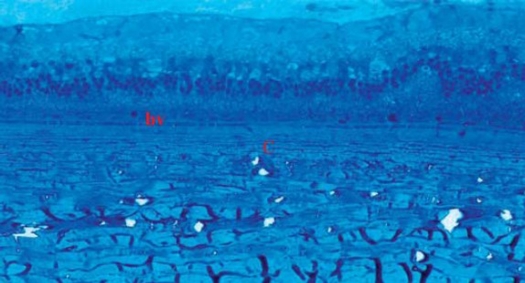
Light micrograph of Avastin treated rabbit eye showing stretched blood vessels with narrow luminae (bv). The surrounding collagen fibers are compact (c), (Toluidine blue × 250).

**Figure 5:**
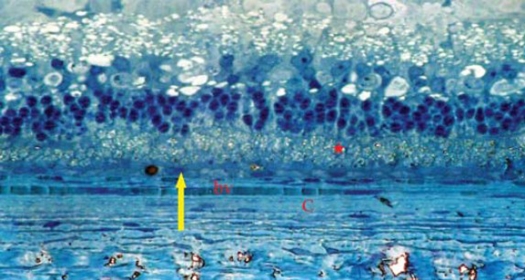
Light micrograph of Avastin treated rabbit eye with higher magnification showing intra-luminal RBCs in a ribbon like arrangement. The surrounding collagen fibers are compact (c). The RPE cells show prominent nuclei (arrow). Mild disruption is seen in the photoreceptor outer segment and vacuolization in ganglion cell layer (arrow) (Toluidine blue × 500).

Light Microscopic Examination: specimens from Avastin group were evaluated in comparison to control eyes. Treated eyes exhibited the same microscopic appearance in most specimens (8/10, 80%). The chorio-capillaris layer showed elongated, stretched monolayer of capillaries with flat, elongated endothelial cell lining (Figs 2, 3). The laminae showed closely packed RBCs arranged in a monolayer with ribbon like shape. The surrounding interstitial tissue showed stretched, elongated, compact collagen fibers (Figs 4, 5).

The RPE cells were tightly adherent to each other with prominent nuclei. The different retinal layers were in concomitance with the control specimens, however mild to moderate disruption of photoreceptor outer segments together with mild vacuolization in the ganglion cell layer were seen (Fig 5).

**Figure 6:**
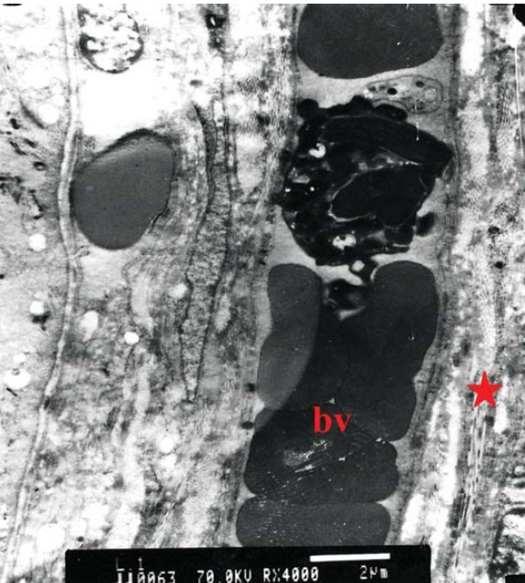
Electron micrograph of control choroid showing blood vessel with average diameter filled with blood elements (bv). The interstitial tissue shows obvious spacing between collagen fibers (red star) (original magnification × 4000).

**Figure 7:**
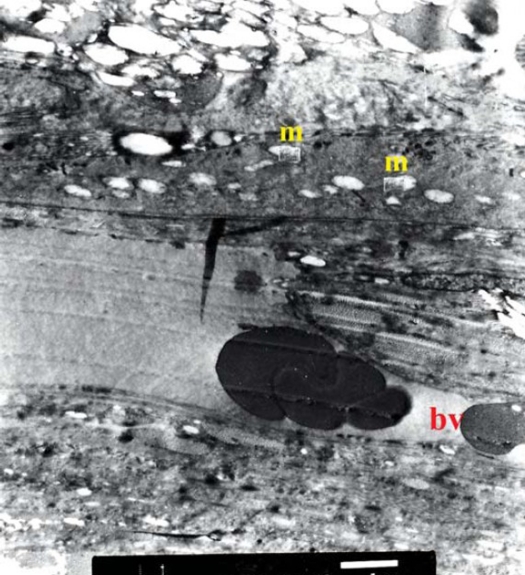
Electron micrograph of control choroid showing RPE cells with active mitochondria (m) (original magnification × 3000).

**Figure 8:**
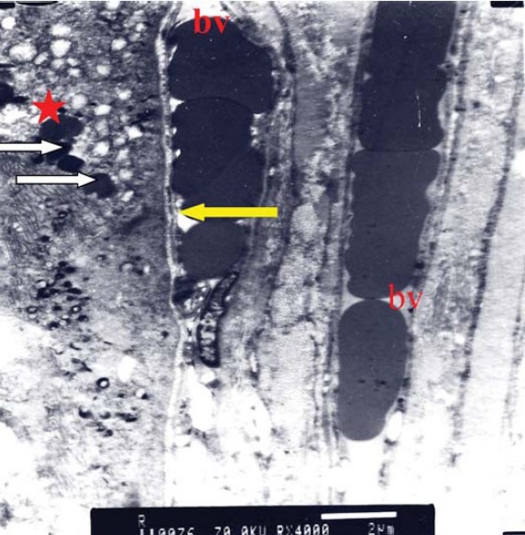
Electron micrograph of treated choroid showing intra-luminal RBCs in a monolayer with ribbon like shape. The endothelial cell fenestration is reduced (yellow arrow). The overlying RPE cell (red star) shows active organelles and prominent melanosomes (white arrow), (original magnification ×4000).

**Figure 9:**
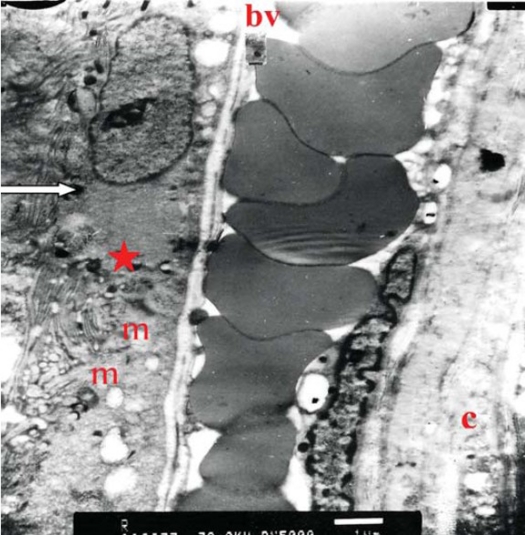
Electron micrograph of treated choroid showing RPE cells (red star) with prominent active mitochondria (m), and endoplasmic reticulum (arrow). The interstitial tissue shows compact collagen fibers (c), (original magnification × 5000).

TEM examination of both control and treated specimens confirmed the findings recorded by LM, however further details were recorded (Figs 6, 7, 8, 9).

The endothelial cell lining of the chorio-capillaris exhibited reduced fenestrations in between the cells (Fig 8). TEM also highlighted the compact lamellae of collagen fibers.

Concerning the RPE cells, there was remarkable increase in number of mitochondria and prominent endoplasmic reticulum. Variable sized melanosomes were also seen (Fig 9).

## Discussion

The histological structure of the rabbit choroid and retina is essentially the same as that of the human choroid. Thickness ranges from 48μ above to 120μ below the optic disc. The chorio-capillaris forms a single layer of fine capillaries.^11^

VEGF is a homo-dimeric glycoprotein. It is a critical regulator of vasculo-genesis and angiogenesis.^12^–^13^–^14^ It is a potent inducer of vascular permeability via multiple mechanisms, including endothelial injury (15), fenestrae formation,^16^ dissolution of tight junctions, and trans-cellular bulk flow.^17^,^18^

In addition to vasculo-genesis and angiogenesis, VEGF may participate in the maintenance of certain vascular systems in the human adult. Specific binding of VEGF is associated with mature vessels in various adult rat tissues, suggesting that VEGF has a function in the maintenance of quiescent vascular endothelium.^19^

Interruption of VEGF production in the retina and RPE in developing animals has been shown to cause serious defects in retinal and choroid development. Elimination of VEGF in adult animals by genetic or pharmacologic means has not been shown to result in changes in retinal or choroid vasculature.^20^–^21^ However, Peters et al in a study of the effect of intravitreal bevacizumab on ultra structures of primate eye; recorded change in the chorio-capillaris in the from of reduction of chorio-capillaris endothelial cell fenestrations.^22^

In a normal eye the RPE secretes VEGF at its basal side, which is required for the maintenance of the chorio-capillaris.^10^ Absence of VEGF causes secondary atrophy of the chorio-capillaris^23^ and results in a loss of endothelial cell fenestrations.^24^

In the non-human primate eye, intravitreal injections of VEGF are capable of triggering a severe diabetic phenotype, including neovascularization of the retina and iris.^25^,^26^

Therapeutic antagonism of VEGF in animal models results in significant inhibition of both retinal and choroid neovasculariztion as well as reduction in vascular permeability.^27^,^28^

In our study, intravitreal injection of Avastin in rabbit eyes resulted in decreased choroid capillaries permeability as suggested by the remarkable decrease in background choroid fluorescence which might be secondary to decreased free passage of dye via chorio-capillaris fenestrae.

Histopathologically, no significant changes were encountered in the retina and choroid of treated eyes by light microscopy.

Several investigators suggested that a single intravitreal injection of bevacizumab at doses up to 5 mg in rabbit eyes dose not result in obvious retinal toxicity. The end point ERG did not have a significant decrease. However, the lack of changes by light microscopy does not exclude possible alterations on a submicroscopic level.^29^–^31^

On the other hand, in our study, TEM sections showed decreased chorio-capillaris permeability augmented by the blockage of fenestrae between chorio-capillaris endothelial cells, as well as compactness of collagen fibers in the stroma as compared to controls. This would be in accordance with the work done by Peters et al.^22^

Another TEM feature was the increase in intracellular content of mitochondria and prominent endoplasmic reticulum of RPE cells reflecting increased activity of the cells which might be a feed back mechanism induced by decreased VEGF concentration around RPE cells.

## Conclusion

Though single intravitreal injection of Avastin does not cause appreciable histological changes in rabbit retina and choroid, yet, it imposes definite effect on chorio-capillaris permeability as evidenced by FFA and ultra-structural changes. Repeated intravitreal injections might alter the hemeostasis of the chorio-capillaris RPE complex.
